# Origin of Mineral Contents of Mineral Springs

**Published:** 1917-08

**Authors:** Felix Von Oefele

**Affiliations:** New York


### Origin of Mineral Contents of Mineral Springs.

DR. FELIX VON OEFELE, New York.

Mineral spring waters contain a varying amount of different mineral constituents in solution. The number of predominant chemical constituents found, including bases and acids, does not exceed a dozen. This definition concerns all the natural existing waters obtainable. Chemically pure water can be produced by the artificial reaction of pure hydrogen and pure oxygen in the chemical laboratory.

Pure water is kept pure with difficulty; it is a colorless, odorless and tasteless fluid and has a high solvent power for many chemical compounds. An almost pure water is the so-called distilled water purified by evaporation and recondensation of the vapors obtained. It is similar to rain water, but still purer. The rain water is distilled by nature and absorbs some vapors and suspensions of the air, from which the artificially and properly distilled water is protected. Water as it falls from the clouds in the form of rain, absorbs particularly, various gases, impurities of the air, so that when obtained in its purest natural state, it contains Oxygen, Nitrogen, Carbonic acid and some times traces of carburated Hydrogen, Sulphurous acid, Nitric acid and Ammonia.

Having fallen upon the earth, the principal part of this water penetrates the soil and rocks of the surface of the earth and it may lose some part of its contents, but takes up various other salts and organic substances from the soil. It reappears earlier or later on the surface of the earth. This reappearing water keeps in solution, more or less material dissolved from the soil and rocks.

If spring or well waters have been evaporated, a small amount of residue is left corresponding to the carried mineral derivatives. It is a small list of mineral compounds found in this average residue. An arbitrary combination and variation of salts by the reporting chemist often seems to indicate to the laymen a larger number of constituents. That is pseudo-scientific caprice, but not natural constitution of the water contents. The common mineral constituents of the waters vary by absolute and relative amount in a very large percentage of springs and wells within narrow limits, and allow the domestic and technical use of these waters. A small percentage exceeds these limits remarkably in quantitative or qualitative or both directions; they show a clear pharmakodynamic effect upon the human organism. These later small percentage is understood in general perception as mineral springs of proper sense.

The world-wide similarity of the majority of average springs and wells is understood from the geological development of the surface rocks of the earth as part of our sun system.

All spring waters having been in contact with different strata of the earth differ from the original rain water holding in solution variable quantities of anorganic compounds, the nature and quantity of which depends upon the geology and petrography of the region. The primary and secondary constituents of the surface of the earth are derived from a uniform thick cover. This consisted of a combination of very few chemical elements. Therefore every spring water contains some carbonic acid, sodium, calcium, chlorine, sulfuric acid and silicon, on account of the original chemical constitution of the earth's surface and the relative solubility of the constituents.

The same constituents are the anorganic matter of the human serum and of all the physiological liquids of animals and plants. Average springs contain then, too, smaller quantities of some rarer constituents, for instance Potassium, Magnesium, Bromine, Iodine, Iron, Aluminum and other ones.

If we still stick to the theory of Laplace for the formation of our sun system, some time ago the entire mass of sun and planets together was a large gaseous sphaeroid with an equatorial diameter exceeding the distance of Neptune to the sun. According to the physical laws governing the gases this sphaeroid contained concentric layers. The elements have been entirely dissociated. The surface layer was formed by Hydrogen. The next gaseous layer must have been Helium, the third Lithium, the fourth Berillium and so on down to Uranium and farther unknown elements exceeding the atomic weight of 238.

Not one of these layers was chemical pure. It was the same condition as that still persisting in our atmosphere which is not exactly divided into a lower part of Oxygen and an upper part of Nitrogen with the small difference of volumic weight 7:8. We have to consider in later parts of the balneology the exhalations of carbonic acid. Where the earth exhales carbon dioxide with the volumic weight of 11 in relation to the above figures 7 and 8, this difference is large enough to create a persistent bottom layer of carbon dioxide. The Grotta del cane in the vicinity of Naples, Italy, is known to contain on the bottom about 8 inches high an air containing about 77% of carbon dioxide. The air of a higher level contains less than 2% carbon dioxide. A light on the bottom is extinguished and a small dog dies within a short time, but human beings in an upright position, are not hurt.

In the neighborhood of highly carbonized springs, particularly in cellars in the district of carbonized springs, the carbon dioxide layer may reach the mouths of men and kill them.

At Bad Neuenahr the director was a tall man and I am a short man. We once were within the fence of the Grosse Sprudel. By some natural circumstances the upper level of concentrated carbon dioxide started to raise and reached my mouth with the characteristic taste. I quickly jumped to a higher step, but the director laughed and went out slowly; he was safer on account of his height. This carbonized spring was thrilled 60 years ago and excludes the general public by a closed iron fence. Before laborers can work there in times of the high tide of the carbon dioxide, the excess of this gas has to be expelled by a certain device. In two different years a small girl and a porter died there of suffocation upon entering the fenced space. The porter died as he stooped to pick a lost ball. To the exactness of a quarter of an inch the boundary between highly concentrated carbon dioxide and respirable air can be given. Where this boundary level touches the vertical surrounding walls, a line of demarcation is visible to the human eyes. The carbon dioxide below this plain is never free of a mixture of oxygen and nitrogen and the air above this plain is never free of carbon dioxide. But this plain is a clear demarcation of the preponderance of constituents.

The physical laws are important in another sense for balneology. The brittle soil of the earth contains organic matter continuously becoming carbon dioxide and besides this water. The water evaporates with a volumic weight below 5 in the above calculation and for this reason shows a tendency to enter the atmosphere and to ascend within it. The carbon dioxide with the weight of 11 can not escape from the soil and becomes absorbed by the circulating water of the soil. Every spring water contains therefore a higher percentage of carbon dioxide than rain, lake or river water. This content of carbon dioxide does not entitle it to the claim of being a carbonized mineral water.

The laws of separation of gas layers are general laws and have been shown important for some properties of mineral waters. We have to keep them in mind for later parts of balneology. There we consider the gaseous layers of the aboriginal sun system. The layers were no separation of chemically pure evaporated elements. Every one contained a good amount of absorptions of others, some times to a great distance. It seems that particularly the electro-negative elements had a high tendency to be absorbed to lower layers and electro-positive ones to higher layers.

For this reason and on account of the thin layer declines from the equator to the poles, covered with lighter elements, hydrogen, helium, etc., are present down to the central sun. On the other hand Neptune, Uranus, Saturn and Jupiter consist principally of the elements of lowest atomic weight.

They are formed from the outermost part of the equatorial surface layers including no part of the deeper layers. I would say, that Neptune is a concentration of Lithium and Beryllium. Uranus shows a decrease of these elements and an increase of Boron and Carbon. Saturn may bear a high excess of Nitrogen and Oxygen. Jupiter may consist principally of Fluorine, Sodium and Magnesium. Of the electro-positive elements the planetoids may contain still an excess of sodium and magnesium and of the electro-negative ones Phosphorus, Sulphur and Chlorine. Mars may approach the earth's composition, but with predominant Calcium. The earth contains principally Iron, Silicium and Oxygen. If Zinc and the elements of rare earths prevail in Venus, its volumic weight may be understood. On this basis Mercury has to contain predominant silver. The heavy elements are present in the sun in a very high temperature and are decreased by heat extension in the average volumic weight of the sun to about the figure of Jupiter. The volumic weight of the moon is still smaller than the earth. The moon has attracted a large quantity of Calcium and Aluminum.

The consolidation of the earth repeated the same process of formation of gas layers as the separation of the planet bodies from the remaining central body, and the formation of the moon from the earth. The solidified surface shows a concentration of the lightest solids. This makes it understood, that compounds of oxygen, aluminium and silicium predominate all over the earth's surface.

Gneiss and Granite were the original cover of the earth. These are rocks with a high degree of free acidity on account of an excess of silicium dioxide. Basalts, lava and other eruptives from deeper strata of the original doughy earth skin approach neutrality. Maybe within the condition that vaporous earth surface was formed, Silicium and Oxygen had already an affinity to form the chemical compound of silicium dioxide, but iron may have been still dissociated to separate atoms in analogy to mercury vapors. Both constituents would have not more difference of volumic weight than Nitrogen and Oxygen up to this time mixed in the atmosphere. Silicium dioxide and iron monatomic may have been the principal part of the aboriginal sun sphaeroid forming the present earth. This would indicate vapors of the volumic weight 28 to 30 measured by diatomic Hydrogen gas of the same condition. The absorption of this original gas layer did not include impurities by elements of a higher atomic weight than 238. The central parties of sun and maybe small admixtures of Venus and Mercury may contain this further continuation of the periodic system of elements.

In the time that the sun system was a gas sphaeroid, the chemical elements had very little or no tendency to form chemical compounds. They were compressible by the pressure of overlasting gases and absorbed the one the other to a small amount as above mentioned for the lower and upper part in the Grotta del cane. Their volumic weights within this compression are still in direct relationship to the molecular weight. This condition of compressed gaseous concentric layers still persists in the sun, as N. Johannsen of Rosebank, N. Y., claims. It forced the separation of the different elements and their simple compounds to strata similar to an onion body. The principal constituents of the earth ball may have been, as explained, Silicium dioxide and iron. They sedimented again and also their admixture of rarer elements, as they reached the liquid and solid condition, where the relationship of volumic weight became strongly changed. Uranium was an original small central nucleus, Thorium and other ones followed in the centre. The surface showed Hydrogen, Helium, Lithium, etc., in small amounts. Nitrogen and Oxygen followed in larger amounts. This arrangement became disturbed, by decreasing temperature, as more and more elements became cooled to a point where the chemical affinity start to be efficient, or the element liquefies or both happen. Most of the elements had become liquid or semi-solid, while the original onion-like arrangement persisted. The new chemical reaction was in efficiency between neighboring shells of elements. Carbon and still more Silicium had the highest affinity to oxygen in the high temperature and pressure. Graphite is present together with Silicium dioxide. Silicium dioxide particularly from the condition of compressed vapor going to a liquid or semi-liquid condition lost very much of its relatively high volumic weight. Its property as acid radical enabled it to stay mixed as acid salt (bisilicate) with basic elements of a lower atomic and volumic weight or of not very much higher atomic weight forming the chemical compounds of the solid surface of the earth. Some of the elements of lower atomic weight persisted as an excess of gases forming the atmosphere.

Small amounts of the heavier elements became impurities of the silicated rocks of the original surface. Other small amounts of compounds of rarer elements were ascendingly pressed in faults and cracks of the solidified surface by the different reactions between surface and contained interior. The metal sulfides often found in veins of the rocks may be principally sublimations of lower gas strata to the surface after first solidification.

The compounds of the original solidified surface became crystals in the slow cooling process. The commonest of these rocks are granite and gneiss with very many variations of size and arrangement of the three constituent kinds of crystals: mica, feldspar and quartz. Later on by decomposition they delivered the principal material for all the surface layers of the earth.

This is the reason that the number of predominant elements of the oldest solidified surface of the earth and of sedimentary rocks is very small. The following geological development of the earth surface was principally shrinking with formation of mountains and valleys and secondary influences of circulating water.

Of the elements with valide affinities air contains 4, sea-water contains 30 elements in dissolved compounds. Rocks contain all the elements known in an irregular distribution over the earth's surface, but the principal part of the surface of the earth contains 8 elements found everywhere in larger or smaller quantities.

Within the crystalline rocks, the average is:

Oxygen 46.3%.
Silicium 29.5%.
Aluminium 8%.
Iron 6.1%.
Calcium 3.7%.
Magnesium 1.4%.
Sodium 2.5%.
Potassium 2.3%.

This restriction of the average surface is due to the above explained development resulting in granite and gneiss to being the real skin or bark of the earth ball.

Iron is really the predominant constituent of meteor stones and seems according to calculated average volumic gravity 5.6 of the complete earth to be the principal part of the interior earth. Some of the rare elements of high atomic weight may become predominant toward the centre of the earth in an amount unexpected from the surface condition.

Water which reappears from the earth, had the greatest chance to dissolve some of the common surface constituents. The compound of sodium and chlorine was the most easily soluble constituent. It became dissolved and almost exhausted from the crystalline rocks in the long geological eras. It has been carried by the circulating water to the sea and stays there retained, if some part of the surface of the ocean is reevaporated. The earth surface is as a rule fairly exhausted of sodium chloride. The percentage of other soluble constituents of the uppermost layers of earth surface is small and the chance of reappearing water to have obtained a supply of very deep strata is slight. We will term these deeper strata juvenile rocks. The surface layers then, too, no longer contain absorbable gases in remarkable amounts. Waters rising from juvenile rocks may be charged with a high amount of gases. The exceptionally small amount of newly formed carbon dioxide of the surface is referred to above.

All the water which reappears out of the earth, was in contact at least with the brittle drift containing traces of carbon dioxide and mostly with some further exhausted layers of rock, and it dissolved there a small amount of the remaining material, derivatives of original gneiss and granite and soluble with difficulty. This is the condition of the majority of springs and wells, which imparts a similarity to the majority of spring and well waters.

In general the common constituents of mineral waters derived from the average constitution of gneiss and granite, we will term invalid or common constituents and other constituents we will call valid or rare ones. An intermediary group is the almost soluble invalid constituents which may obtain an inexpected higher solubility by physical means, particularly by the colloid condition.

The rain water penetrating the surface tends to reach the deepest point down to impermeable strata. The surface of these strata is curled as a rule with some average decline in some direction. There the water follows the deepest paths and corrodes its ways forming continuous underground veins in an arrangement similar to a surface river system. On the contrary the underground waters run very slowly. By the long periods of existence of these veins, the already dissolved material amounts to so much that the surface above such underground veins shows clear depressions noticeable to an experienced eye. Where, by a hilly decline, the surface of earth reaches a deep point of such a current and where, moreover, the underlying impermeable stratum does not allow a deeper penetration of the running water, the water reappears continuously running to the surface. Often such a spring in the original condition is still covered by a small amount of brittle drift or it may form a swamp of its vicinity.

It is a matter of experience to learn from the configuration of the surface the location and direction of underground water veins. I obtained this experience in the first years of my medical practice in farm regions. My main teacher was Aufschlaeger, a blacksmith at Simbach in Bavary, not a scientific, but a practical man. This experience is very important to find out places suitable for well digging. While practicing in Lower Bavary, I was consulted in a large circuit for farmers water supply. For decades I have done no more practical work in this field.

---

**Non-Protein N. and Urea in Maternal and Foetal Blood at Time of Birth.** J. Morris Slemons and W. H. Morris (sic), Bull. Johns Hopkins Hosp., Dec. 1916, summarize as follows:

1. In 35 normal obstetrical patients at the time of birth the average rest-nitrogen in the maternal blood was 25.2 mg. per 100 cc. (extremes 18.5-33.5 mg.) ; in the fetal blood the average was 24.9 mg. (extremes 19.34.2 mg.).

2. In 16 normal patients the average quantity of urea-nitrogen in the maternal blood was 10.5 mg. per 100 cc. (extremes 8.4-14 mg.) ; in the fetal blood the average was 10.4 mg. (extremes 7.9-13.5 mg.).

3. The urea-nitrogen represented 44 per cent of the rest-nitrogen in the maternal and 45 per cent in the fetal blood.

4. The same concentration of urea in both circulations indicates that this substance passes through the placenta by diffusion.

5. Before this is said of the rest-nitrogen, each of its constituents should be studied separately, though it appears that equalization of the rest-nitrogen is normally maintained on the two sides of the placental partition.

6. Complications accompanied by an increase of urea in the maternal blood—toxaemias of pregnancy, syphilis, decompensated heart lesions, and others—are also attended with a corresponding increase in the fetal blood-urea. Pathological cases thus confirm the conclusion that urea diffuses through the placenta.

7. The administration of chloroform during pregnancy causes alterations first in the fetal and later in the maternal blood. Primarily the fetal blood-urea is increased. Prolonged anaesthesia causes a moderate increase in the rest-nitrogen of both circulations.

8. Asphyxia dependent upon impairment of the fetal heart-action is attended with a notable increase in the urea of the fetal blood. In cases of still-birth this generally represents 60 to 85 per cent of the rest-nitrogen.

